# From community to policy: a frontline model for dementia assessment and brain health

**DOI:** 10.3389/frdem.2026.1821709

**Published:** 2026-06-16

**Authors:** Kyle DeDecker, Ngozi Iroanyah, Adam Morrison, Julie Datta, Ibukun-Oluwa Omolade Abejirinde, Caitlin R. Siu, Christina Stergiou-Dayment, Shawn Paron, Christa M. Studzinski

**Affiliations:** 1 Alzheimer Society of Ontario, Toronto, ON, Canada; 2 Institute for Better Health, Trillium Health Partners, Mississauga, ON, Canada; 3 Dalla Lana School of Public Health, University of Toronto, Toronto, ON, Canada; 4 Institute for Health System Solutions and Virtual Care, Women’s College Hospital, Toronto, ON, Canada; 5 Ontario Brain Institute, Toronto, ON, Canada

**Keywords:** co-creation, cognitive assessment, community, dementia, evaluation, implementation, neurotechnology, registry

## Abstract

Globally, dementia prevalence is projected to rise from approximately 55 million people in 2025 to 139 million by 2050. In Ontario, prevalence is estimated to reach 756,100 people by 2050—an increase of 202% since 2020—placing increasing strain on care partners, families, and health systems (Alzheimer Society of Canada, 2022). Throughout Ontario and Canada more broadly, existing pathways for cognitive screening, assessment, and diagnosis are constrained by limited primary care capacity, prolonged specialist wait times, and inequitable access, leaving many individuals without timely assessment or connection to supports. To help address these gaps, the Alzheimer Society of Ontario (ASO) and the Ontario Brain Institute (OBI) leveraged an existing community-delivered cognitive assessment process and enhanced it through the co-development of a provincial dementia registry—the first of its kind in Canada. This novel integrated approach embeds structured cognitive and functional assessment within community organizations, shares assessment results with primary care, and links standardized data to a provincial registry infrastructure. Together, this supports timely identification of cognitive concerns and enables the collection of a standardized minimum dataset—including cognitive, functional, demographic, and care-related information—across diverse populations. The dementia registry can serve as infrastructure to evaluate dementia-related innovations (including digital cognitive-screening tools) in real-world settings and support evidence generation to inform service planning and policy development, while strengthening earlier connection to supports and more coordinated care pathways for individuals and their care partners. This approach is aligned with emerging regulatory emphasis on real-world evidence (U.S. Food and Drug Administration, 2018; European Medicines Agency, 2021; Health Canada, 2019) and with Ontario’s Improving Dementia Care in Ontario Act (2024), illustrating how co-designed, frontline-led approaches may bridge community needs and health system decision-making. Together, this work lays the groundwork for a scalable framework to advance equitable, person-centred dementia care in Ontario and beyond.

## 1 Introduction

Dementia is among the most pressing and complex health system challenges of the twenty-first century (Alzheimer Society of Canada, 2022). It is the seventh leading cause of death worldwide and one of the major causes of disability and dependency among older adults (World Health Organization, 2017). Globally, the prevalence of dementia is projected to rise from approximately 55 million people in 2025 to 139 million by 2050, driven largely by population aging and increased longevity (Alzheimer’s Disease International, 2025). In Canada, more than 750,000 people are currently living with dementia, a number expected to nearly triple by 2050 (Alzheimer Society of Canada, 2022). In Ontario alone, prevalence is estimated to reach 756,000 people by the same year—an increase of 202% since 2020—placing significant strain on care partners, families, and health systems across the province (Alzheimer Society of Canada, 2022). These trends underscore the growing urgency of timely identification, coordinated care pathways, and system-level planning tailored to Ontario’s evolving needs (Alzheimer Society of Canada, 2022; Government of Canada, 2019).

In Ontario, as in many jurisdictions, existing care pathways are ill-equipped to meet the demand of more than 350,000 people currently living with dementia. Access to primary care remains inequitable, and short appointment times often limit opportunities to conduct structured cognitive screening or follow-up assessments in routine practice (Canadian Medical Association, 2023). For individuals referred onward, wait times to see a specialist frequently exceed two years (Ontario Council of Hospital Unions (OCHU), 2026). And yet, these pressures are not experienced evenly: rural and remote communities, First Nations, Inuit, and Métis communities, newcomers to Canada, and individuals facing linguistic, cultural, or socioeconomic barriers encounter additional challenges that further delay assessment and engagement with the care they need (Alzheimer Society of Canada, 2024). These pressures are intensifying as disease-modifying therapies and emerging diagnostics increase the need for earlier identification, alongside growing regulatory emphasis on real-world evidence to inform system-level decision-making (US Food and Drug Administration, 2018; European Medicines Agency, 2021; Health Canada, 2019).

Bill 121, Improving Dementia Care in Ontario Act, 1st Sess, 43rd Parl, 2024 reflects a policy commitment to strengthen early detection and enhance coordination across the dementia care continuum. Yet, despite this legislation, there remains no provincial infrastructure to support the systematic collection of a standardized minimum data set for people living with dementia in community settings, including cognitive screening scores alongside demographic and care-related information, linked to real-world data systems. As a result, dementia-related needs are often identified late, inconsistently documented, or not captured in ways that meaningfully inform system planning, service coordination, and policy development.

## 2 System gaps and the case for registry-enabled infrastructure

### 2.1 Diagnostic and access constraints in Ontario

Obtaining a timely dementia diagnosis in Ontario remains limited by structural features of the healthcare system rather than by lack of demand. Structured cognitive screening requires time, follow-up, and continuity—resources often constrained within current primary care models.

When cognitive concerns are brought to primary healthcare providers, bottlenecks often arise due to limited appointment time, competing clinical priorities, variability in access to structured screening tools, and constrained specialist referral capacity. These factors contribute to delayed diagnosis and fragmented access to care. In Ontario, specialist capacity has not kept pace with demand; when individuals are referred to specialized services such as memory clinics and geriatric specialists, delays are prevalent [Ontario Council of Hospital Unions (OCHU), 2026]. Because of this, people living with dementia and their care partners may experience prolonged distress and difficulty accessing appropriate supports. Early education is becoming increasingly important as recent research suggests that 45% of dementia cases may be prevented by addressing modifiable risk factors (e.g., hearing and vision loss, high cholesterol, social isolation, physical inactivity, etc.) (Livingston et al., 2020). However, opportunities for early education, risk reduction, care planning, or other interventions are often missed.

These challenges are magnified for communities already facing inequitable access to care. For example, rural and remote regions contend with healthcare provider shortages and travel barriers; Indigenous communities continue to face the impacts of historical and ongoing inequities, including experiences of culturally unsafe care and mistrust of healthcare systems; and newcomer communities may encounter linguistic barriers, stigma, and limited familiarity with available services. As a result, pathways to cognitive screening, assessment and diagnosis are highly variable across the province, shaped as much by geography and social context as by clinical need (Alzheimer Society of Canada, 2024).

### 2.2 Community organizations and missed opportunities

Community-based Alzheimer Societies and related organizations across Ontario play an important role in supporting people living with dementia and their care partners. They provide education, counselling, and connections to local health and social care resources, often serving as the first point of contact when cognitive concerns arise. Despite this, community organizations have historically been positioned downstream of diagnosis rather than embedded within assessment pathways themselves. This differentiation has limited opportunities to identify concerns in a timely manner and connect individuals to supports early on in their dementia journey.

### 2.3 The need for real-world data and registry infrastructure

Equally significant is the lack of real-world data. Healthcare system planning and policy development rely heavily on administrative and epidemiological data; however, these sources rarely capture social or contextual information, and they provide little insight into equity-related gaps in access to care, resulting in limited insight into how, where, and when people first seek assessment or support. Without structured data on screening and navigation, it is difficult to evaluate the impact of new policies, identify unmet needs, or plan for future demand. This absence of real-world evidence constrains the ability of policymakers to design responsive, equitable dementia strategies aligned with lived experience.

A dementia registry is a valuable tool for quality improvement and system planning. Traditionally, registries have focused on diagnosed populations within clinical settings, emphasizing disease and treatment patterns. While these registries provide important insights, they capture a limited segment of the dementia care continuum (Garland et al., 2025).

Internationally, several dementia registries exist, but most capture individuals later in the pathway and offer limited insight into the point at which concerns first emerge in community settings. Some, such as those in Sweden and the United Kingdom, generate data but largely capture later stages of the care pathway (Skoog et al., 2014; National Health Service (NHS) England, 2025; Garland et al., 2025). Few initiatives embed standardized screening within trusted community organizations while simultaneously generating structured real-world data from frontline delivery. This gap represents both a challenge and an opportunity in Ontario and Canada more broadly.

A registry linked to community-based cognitive screening offers a different perspective. By capturing data at the point where concerns first emerge, such a registry can document care pathways that precede diagnosis, including functional changes, access to supports, and social context. This enables analysis of who is receiving assessments, who is not, and where barriers persist across diverse populations. It also lays the groundwork for timely access to appropriate supports.

A dementia registry embedded within a community-based screening model can also hold other important functions: drive quality improvement and best practices by examining how assessment pathways function in real-world settings; provide an infrastructure for evaluating dementia-related innovations (such as digital cognitive screening tools) across diverse populations rather than clinical groups; and also generate evidence to inform service planning and policy development.

## 3 The Ontario community-based screening and registry model

In response to the system gaps outlined above, the Alzheimer Society of Ontario (ASO) and the Ontario Brain Institute (OBI) developed a community-based cognitive screening and dementia registry model designed to strengthen early identification, improve coordination with primary care, and generate real-world data for system learning. The model integrates trusted community delivery with standardized data capture and provincial governance infrastructure. Together, these elements establish a structured entry point into dementia care while supporting longitudinal population-level insights.

### 3.1 Alzheimer Society of Ontario (ASO) and the Ontario Brain Institute (OBI) partnership

The partnership between ASO and OBI is foundational to the dementia registry model’s design and implementation. ASO contributes frontline expertise, community partnerships, and province-wide reach to people living with dementia. OBI contributes data governance and infrastructure, research and evaluation expertise, and experience building large-scale datasets with standardized data elements.

Together, this partnership bridges community delivery and scientific rigour by linking existing community-based cognitive screening and functional assessment pathways to standardized data capture and registry infrastructure. This collaboration supports the collection of a minimum dataset in real-world community settings—an approach that neither organization could achieve independently. The result is a model grounded in lived experience that is positioned to inform ongoing quality improvement and evidence-informed decision-making in Ontario.

### 3.2 Registry-enabled community assessment infrastructure in Ontario

Ontario’s community-based cognitive screening pathway serves as an entry point for individuals and families seeking connections to support. This model is distinct in that it links these trusted community workflows to a provincial dementia registry with rigorous governance and consent processes, supporting transparency and ethical stewardship of data. The innovation lies in the systematic capture and governance of standardized data arising from community-delivered assessment processes.

During the roll-out phase, individuals are reached through multiple entry points, including referrals from primary care and other health and social service providers, as well as self-referral and outreach through community-based Alzheimer Society programs and partner networks. Patient engagement is primarily facilitated through existing community-based service delivery models, where organizations already conduct cognitive assessments and maintain ongoing outreach. The registry is integrated into routine clinical practice within these settings and offered to clients during standard care interactions, supporting early identification while leveraging established access points and trusted relationships, and reducing reliance on specialist-led pathways.

By integrating registry infrastructure into established community workflows, this approach enables the structured collection of a standardized minimum dataset at the point where cognitive concerns are first discussed. This minimum dataset includes cognitive and functional measures alongside key demographic, contextual, and care-related variables that illuminate real-world trajectories across the dementia continuum. Capturing this information in a consistent format enables examination of patterns of access, barriers to follow-up, service utilization, and variation across regions and populations.

Importantly, this infrastructure does not replace clinical diagnosis or primary care processes. Rather, it supports timely identification of cognitive concerns and facilitates clearer coordination with primary care providers and specialists, while enabling longitudinal data capture to support quality improvement, service planning, and evaluation over time.

## 4 Model design and core components

### 4.1 Person- and care-partner-centred design

The model emphasizes clarity and comfort for people with cognitive concerns and their care partners. Assessments are conducted in community-based, non-medicalized environments designed to reduce fear and stigma associated with cognitive decline. Assessment results are then communicated by providers in a way that supports shared decision-making and next steps.

### 4.2 Community-embedded data capture

Participating community organizations collect standardized information alongside contextual variables that reflect lived experience. Participating community organizations collect a standardized core set of data elements using assessment approaches selected for appropriateness to scope of practice, setting, and the linguistic and cultural context of the population served. Wherever possible, culturally and linguistically validated tools are prioritized, and any local adaptation is undertaken within a harmonized registry framework to preserve comparability across sites.

In addition to this data, extended elements may be incorporated to reflect local context, including geographic factors, language considerations, and barriers to access. This flexibility allows the registry to preserve standardization while remaining responsive to community needs. Future development aims to strengthen the capture of care partner data, recognizing that global reviews of dementia registries have identified caregiving information as inconsistently collected despite its importance for understanding care trajectories and system burden (Garland et al., 2025).

### 4.3 Informatics platform and data governance

Registry infrastructure is provided by OBI through Brain-CODE (Stuss, 2014). Brain-CODE is OBI’s large-scale neuroinformatics platform, developed to support the collection, storage, federation, sharing and analysis of different data types collected from several different research programs that study a broad span of people living with brain disorders across Canada. The technical and governance features of Brain-CODE have been described previously (Vaccarino et al., 2018; Lefaivre et al., 2019; Behan et al., 2023). The platform ensures that the registry data adhere to FAIR principles—specifically that the data are Findable, Accessible, Interoperable, and Reusable (Behan et al., 2023). For example, OBI has developed a Data Quality Framework (DQF) to support quality data collection and generation.

Brain-CODE operates on a robust governance structure supported by participant consent and ethics board approvals. These processes govern which data can be collected, uploaded, de-identified, and shared on Brain-CODE. While all participating sites begin with standardized consent language, there are cases where the language is modified, to better reflect the needs of a particular community.

The registry is voluntary and based on consent with clear communication regarding data use, privacy protections, and options for withdrawal. Data are collected and stored using coded identifiers within a secure environment, and access is governed through defined oversight and review processes. Brain-CODE’s governance structure emphasizes ethical stewardship and appropriate use of data to support quality improvement and research while maintaining public trust and alignment with provincial and national privacy standards.

### 4.4 Co-design and advisory structure

Assessment workflows and associated registry processes were co-designed with people living with dementia and care partners through advisory roles, consultation processes, and iterative feedback. The dementia registry is supported by a multidisciplinary Advisory Group that provides strategic guidance to ensure scientific excellence, clinical relevance, and meaningful community engagement. Building on the leadership of ASO and the technical infrastructure of OBI’s Brain-CODE platform, the Advisory Group includes geriatricians, neurologists, primary care physicians, researchers, industry partners, care partners, disease registry experts, and First Nations representatives. This diverse composition ensures the Registry remains inclusive and representative, grounded in real-world practice, and responsive to the priorities of people living with dementia and their communities.

Lived experience informed decisions related to assessment workflows, consent processes, and communication of results. Co-design ensures that the model reflects not only clinical considerations, but also emotional and cultural realities encountered by individuals at the point when concerns first arise. Co-created approaches are increasingly recognized as important for promoting equity and ensuring that services reflect the diverse realities of people living with dementia (Alzheimer Society of Canada, 2024).

## 5 The dementia registry as enabling infrastructure

### 5.1 Longitudinal data and linkage

Participants may consent to longitudinal follow-up, enabling repeated data collection to examine change over time and trajectories across diverse populations and care settings. Where consent is provided, encrypted health card identifiers permit secure, privacy-compliant linkage to provincial administrative health databases, including hospitalizations, emergency department visits, physician billings, prescription drug claims, long-term care admissions, and vital statistics.

Linkage to routinely collected administrative data enhances the scientific value of the registry by enabling longitudinal ascertainment of health service utilization, clinical outcomes, comorbid conditions, and mortality across the continuum of care. It reduces reliance on self-report, minimizes loss to follow-up, and improves completeness of outcome measurement while avoiding additional participant burden. At a systems level, linkage supports population-level analyses of care trajectories, health system costs, and patterns of access and equity, strengthening the registry’s capacity to generate generalizable evidence to inform clinical practice, health system planning, and dementia policy.

### 5.2 Supporting evaluation of emerging diagnostics and therapies

Where feasible and consented, the registry supports longitudinal follow-up to evaluate emerging approaches such as blood-based biomarkers (BBMs), digital cognitive assessments (DCAs), and disease-modifying therapies (DMTs). Repeated measures enable examination of how these tools perform within community-linked pathways by relating results to longitudinal changes in cognition and service utilization. This may clarify their clinical utility across screening, diagnosis, prognosis, monitoring, and treatment decision-making. At a systems level, follow-up data support evaluation of referral patterns, service use, and unintended consequences, including inequities in access or increased burden on specialized services.

### 5.3 Enabling system learning and planning

Importantly, longitudinal registry data can help address key evidence gaps that are not easily answered through clinical trials alone, including long-term outcomes. This aligns the registry as a foundational platform to inform policy decisions and future investment in diagnostics and therapies.

The registry is positioned to collect harmonized data across multiple assessment pathways to help build real-world evidence to better understand the dementia care pathway across Ontario. This data can be used to identify gaps in current dementia care streams and enable quality improvement in the healthcare system through policy or priority shifts.

## 6 Implementation

### 6.1 Integration with primary care and community services

Implementation began in March 2025 and has focused on feasibility within three communities. Community-based Alzheimer Society staff receive standardized training in the administration and scoring of cognitive and functional assessment tools, informed consent procedures, and registry-related workflows. Training includes certified MoCA Cognition modules (with required certification and biennial recertification) and Cognitive Assessment Tools (CAT) workshops delivered by geriatric experts through the Central Regional Geriatric Program in collaboration with McMaster University. Training is provided by regulated health professionals and senior program staff with expertise in dementia care, with ongoing supervision embedded in implementation to ensure adherence to evidence-based practice.

Assessment results are communicated to primary care providers, who retain responsibility for clinical interpretation, follow-up, diagnosis, and referral decisions. This integration maintains primary care as the medical home for ongoing care and management.

Concurrently, consented assessment data are captured within a secure digital environment. This parallel process allows routine care interactions to generate structured, consented data without altering clinical roles or replacing established diagnostic pathways. This approach embeds registry-enabled data capture within existing workflows, minimizing disruption to clinical service delivery.

### 6.2 Early reflections from pilot implementation

Early implementation experience suggests that embedding the registry within community-based assessment pathways supports acceptability and timely connection to supports compared with referral-dependent models. Participants are offered the opportunity to contribute structured data reflecting their experiences and needs. While the registry does not coordinate care, it establishes infrastructure for generating real-world evidence to inform service planning and quality improvement.

Challenges requiring ongoing attention include staff capacity in high-demand settings to review and obtain informed consent for registry participation in addition to routine clinical consents, as well as constraints on time available for comprehensive confidentiality discussions. There is also variation in digital literacy among both staff and participants, and a continued need to adapt processes to support diverse linguistic and cultural contexts. These early insights are informing iterative improvements to better integrate existing community-based assessment processes with the dementia registry.

### 6.3 Sustainability and scale considerations

To scale and expand registry participation through these community linkages, additional funding is required. Although community-based assessments are delivered at lower cost than primary or specialty care, they are not currently covered under Ontario’s universal healthcare system. Alignment with ongoing government priorities provides an important opportunity to advocate for sustainable investment to support broader implementation.

## 7 Policy and health system implications

The community-based cognitive screening model and linked dementia registry described here have implications that extend beyond program-level innovation. Together, they suggest a reorientation of dementia care pathways toward more timely identification of cognitive concerns, improved equity, and better cohesion among frontline community services, primary and specialized care, and policy decision-making.

### 7.1 Person-centred pathways

Embedding standardized cognitive and functional assessments within trusted community organizations creates an accessible, person-centred entry point into the dementia care continuum. By integrating assessment results with primary care, the model strengthens coordination across settings while supporting earlier education and navigation without increasing pressure on clinicians.

### 7.2 Equity and access implications

The model also carries important equity implications. By embedding screening within community settings, the pathway may reduce geographic, cultural, and logistical barriers that contribute to delayed diagnosis in underserved populations. Standardized data capture enables examination of access patterns across geography, sociodemographic characteristics, and care pathways, supporting early identification of disparities or unintended consequences. This transparency strengthens the capacity of the system to respond to inequities proactively rather than retrospectively.

Yet, implementation of this model must contend with factors that may limit its equity impact in practice. Digital limitations, for one—such as scant access to devices or poor digital literacy—may hamper participation in digitized screening and registry processes, particularly in rural and remote areas. In fact, we have seen this occur with our first attempt at testing a digital cognitive assessment. The learnings from this project will be reported elsewhere and informed how we approach testing digital innovations. Secondly, language barriers and varying levels of health literacy may also affect individuals’ understanding of assessment results and the informed consent process, especially among newcomer populations. As well, historical and ongoing experiences of culturally unsafe care contribute to mistrust of health and data systems among some First Nations and Indigenous communities, which may influence willingness to participate in this project despite robust governance frameworks. Lastly, the use of standardized cognitive assessment tools introduces challenges related to cultural validity, even where efforts are made to prioritize culturally adapted instruments.

To address these challenges, the model incorporates ongoing co-design and advisory input from people with lived experience, including representation from Indigenous and equity-deserving communities, as well as iterative adaptation of consent processes and assessment tools. Flexible pathways—including non-digital options, for example—and partnerships with community organizations are central to supporting culturally safe and accessible implementation. Importantly, the registry itself enables monitoring of participation and access patterns across populations, allowing disparities in uptake to be identified early and addressed through continuous quality improvement.

### 7.3 System sustainability scalability

The registry supports population health management by generating longitudinal, real-world data on early cognitive concerns, service navigation, and outcomes. These insights can inform resource allocation, workforce planning, and policy prioritization. While developed in Ontario, the principles of community-based delivery, co-design, standardized assessment, and real-world data generation are transferable to other jurisdictions seeking to strengthen dementia surveillance and care.

## 8 Evaluation and continuous learning

Ongoing and planned evaluation is guided by the Canadian Network for Digital Health Evaluation, (2022) (CNDHE) framework and a developmental evaluation approach, using a learning health system orientation. Evaluation examines feasibility, acceptability, implementation fidelity, and equity-related access patterns, alongside early signals of health system impact. Outcomes will be assessed in alignment with the “quintuple aim” (Nundy et al., 2022), including patient and care-partner experience, population health, costs, provider experience, and equity. Findings will inform iterative refinement of the model and support evidence-informed decisions regarding sustainability and scale.

Equity-related access patterns will be examined across entry points, including variation by geography, sociodemographic characteristics, and care pathways, to identify potential disparities or unintended consequences early in implementation. This multi-setting lens enables a more comprehensive understanding of who is being reached, where gaps persist, and how different system components interact.

Where data permit, the evaluation will explore early signals of health system impact, including changes in referral patterns, diagnostic timelines, care coordination, and service utilization across community and clinical contexts. Quantitative analyses will be complemented by qualitative insights from providers, researchers, people living with dementia, and care partners to support interpretation and contextualization of findings.

## 9 Conclusion

This article describes a community-based cognitive screening model linked to a provincial dementia registry that responds to longstanding gaps in access, equity, and system coordination within dementia care in Ontario, Canada. By embedding standardized assessment within trusted community organizations and pairing it with real-world data infrastructure, the model reframes how and where cognitive concerns are first identified, and how individuals are supported at critical moments in the dementia journey.

As dementia prevalence continues to rise, the consequences of delayed assessment and fragmented pathways become increasingly profound. Continued investment, partnership, and policy alignment will be essential for sustaining and scaling this infrastructure. Community-based models that centre lived experience while generating actionable, real-world evidence will be critical to ensuring that earlier identification leads not only to timely diagnosis but to earlier and more effective care.

## Data Availability

The original contributions presented in the study are included in the article/supplementary material, further inquiries can be directed to the corresponding author.
